# New Frontiers of Anaerobic Hydrocarbon Biodegradation in the Multi-Omics Era

**DOI:** 10.3389/fmicb.2020.590049

**Published:** 2020-11-16

**Authors:** Krisztián Laczi, Ágnes Erdeiné Kis, Árpád Szilágyi, Naila Bounedjoum, Attila Bodor, György Erik Vincze, Tamás Kovács, Gábor Rákhely, Katalin Perei

**Affiliations:** ^1^Department of Biotechnology, University of Szeged, Szeged, Hungary; ^2^Institute of Biophysics, Biological Research Centre, Szeged, Hungary; ^3^Institute of Environmental and Technological Sciences, University of Szeged, Szeged, Hungary; ^4^Department of Biotechnology, Nanophagetherapy Center, Enviroinvest Corporation, Pécs, Hungary

**Keywords:** anoxic biodegradation, hydrocarbon-degrading microorganisms and microbial communities, catabolic pathways, high throughput technologies, functional genomics/metagenomics

## Abstract

The accumulation of petroleum hydrocarbons in the environment substantially endangers terrestrial and aquatic ecosystems. Many microbial strains have been recognized to utilize aliphatic and aromatic hydrocarbons under aerobic conditions. Nevertheless, most of these pollutants are transferred by natural processes, including rain, into the underground anaerobic zones where their degradation is much more problematic. In oxic zones, anaerobic microenvironments can be formed as a consequence of the intensive respiratory activities of (facultative) aerobic microbes. Even though aerobic bioremediation has been well-characterized over the past few decades, ample research is yet to be done in the field of anaerobic hydrocarbon biodegradation. With the emergence of high-throughput techniques, known as omics (e.g., genomics and metagenomics), the individual biodegraders, hydrocarbon-degrading microbial communities and metabolic pathways, interactions can be described at a contaminated site. Omics approaches provide the opportunity to examine single microorganisms or microbial communities at the system level and elucidate the metabolic networks, interspecies interactions during hydrocarbon mineralization. Metatranscriptomics and metaproteomics, for example, can shed light on the active genes and proteins and functional importance of the less abundant species. Moreover, novel unculturable hydrocarbon-degrading strains and enzymes can be discovered and fit into the metabolic networks of the community. Our objective is to review the anaerobic hydrocarbon biodegradation processes, the most important hydrocarbon degraders and their diverse metabolic pathways, including the use of various terminal electron acceptors and various electron transfer processes. The review primarily focuses on the achievements obtained by the current high-throughput (multi-omics) techniques which opened new perspectives in understanding the processes at the system level including the metabolic routes of individual strains, metabolic/electric interaction of the members of microbial communities. Based on the multi-omics techniques, novel metabolic blocks can be designed and used for the construction of microbial strains/consortia for efficient removal of hydrocarbons in anaerobic zones.

## Introduction

Various hydrocarbon (HC) compounds, derived from crude oil (n-alkanes; other aliphatics; mono-, di- and polyaromatic compounds; heterocyclic aromatics), are the most abundant hazardous organic wastes which are mostly released during the extraction processes, drilling of wells, transportation and unsuitable storage of oil, even in the immediate vicinity of filling stations (Lim et al., 2016; Bacosa et al., 2018; Xu et al., 2018). Usually, these pollutants are mixtures of various compounds including linear, branched and cyclic alkanes, monoaromatic and polyaromatic molecules. In the case of land contamination, oil can migrate into the soil and adsorb to its particles, resulting in reduced soil quality. Hydrocarbons, reaching the groundwater, can spread, causing immense contamination (Xu et al., 2018). The effect of spilled oil on the marine environment is also immensely remarkable (Overholt et al., 2016). Some of the oil sinks to the bottom of the sea, endangering deep-water organisms (Lim et al., 2016). Due to wind-induced waves, oil breaks down into small drops, forming an emulsion in the waterbody, whereas oil spills on the water surface block the oxygen transfer/flow to the pelagic organisms of the sea. The challenge of oil removal from the environment ensued from the diverse composition and hydrophobic properties of the contaminant and weathering effects, as well (Jiang et al., 2016; Lim et al., 2016; Ławniczak et al., 2020).

In an ecosystem, microbes have well-known roles in the conversion of hydrocarbons. Their extensive metabolic activity makes the inexpensive and efficient cleanup of contaminated sites possible. The organic pollutant is commonly utilized as an energy and nutrient source in metabolic processes. Bioconversion of contaminants can take place in either aerobic or anaerobic environments (Meckenstock et al., 2015; Bacosa et al., 2018).

By the end of the 20th century, in many remediation technologies, pure microbial cultures or simple consortia isolated by classical isolation methods have been applied for removal of contaminants, such as hydrocarbons (Löffler and Edwards, 2006). However, it has been recognized that most of the bacteria living in our environment can not be studied by the classical cultivation methods (Amann et al., 1995; Oberhardt et al., 2015). It was frequently observed that a process operating optimally under laboratory conditions did not work in the field and vice versa. The background of these facts was examined, and among others, the effect of environmental factors, horizontal gene transfer, metabolites or other unknown components, derived from the partner microbes, were suggested to be responsible for these phenomena (National Research Council, 1993). In the absence of modern, new generation molecular biological methods, it was tough to resolve the contradictory results obtained in laboratory conditions and on-site experiments.

It is well-known that many properties of conventionally isolated microorganisms in their original environment might be overlooked, which may be owing to the targeted isolation methods. On the other hand, an isolated microbe as monoculture might be inefficient degrader in the lab since its essential synergistic partners were removed during the isolation process (François et al., 2016). Therefore, approaches capable of investigating the processes at the system level have been necessary.

Advanced molecular technologies allow us to disclose metabolic functions, routes, interactions and networks; therefore, their application in biodegradation might revolutionize the bioremediation technologies (Lovley, 2003).

Aerobic biodegradation of aliphatic and aromatic compounds (mono- and polycyclic) has been prominent and documented in several studies (Brzeszcz and Kaszycki, 2018; Kuang et al., 2018; Révész et al., 2018; Xu et al., 2018; Mejeha et al., 2019; Steliga et al., 2020). The microorganisms use molecular oxygen as a terminal electron acceptor under aerobic conditions. Molecular oxygen functions as a trigger component in the activation mechanisms. Oxygenases (monooxygenases and dioxygenases), which transfer one or two oxygen atoms onto the substrate, have a central role in the oxic bioconversion of hydrocarbons (Karigar and Rao, 2011; Hua and Wang, 2014; Bacosa et al., 2018).

It is common knowledge that several microorganisms can utilize aromatic and aliphatic hydrocarbons for their growth under anoxic conditions. This topic was reviewed several times in the last decade (Carmona et al., 2009; Fuchs et al., 2011; Rabus et al., 2016). The identification and characterization of microorganisms capable of anaerobic hydrocarbon mineralization have been a major challenge for researchers because most of these microorganisms exist in a consortium. They are usually not culturable by the classical methods; it is true, especially for the methanogenic members of the hydrocarbon-degrading communities (Kleinsteuber et al., 2012; Laso-Pérez et al., 2019). Even so, a number of research groups have recently published their findings regarding HC-degrading microorganisms in sediments or subsurfaces under limited oxygen conditions (Yang et al., 2016; Bacosa et al., 2018; Espínola et al., 2018; Roy et al., 2018; Sperfeld et al., 2018; Liu et al., 2019; Marić et al., 2019; Miller et al., 2019; Pilloni et al., 2019; Song et al., 2019; Sun and Kostka, 2019; Li et al., 2020; Révész et al., 2020). Several recent review articles have summarized the microorganisms and metabolic pathways involved in the anaerobic hydrocarbon biodegradation. Various microbes, metabolic pathways linked to different redox compounds might coexist in the environment (Boll et al., 2014). Based on an interdisciplinary collaboration, pure and enriched cultures of bacteria and their key enzymatic reactions involved in anaerobic hydrocarbon degradation were reviewed (Rabus et al., 2016). This synopsis review (referring 14 other reviews on the comprehensive work of a large consortium about anaerobic hydrocarbon degradation) primarily focused on the characterization of toluene-activating benzylsuccinate synthase, phylogenetic classifying of alkyl-/arylalkylsuccinate synthases, stereochemical and co-metabolic insights into n-alkane-activating (methylalkyl)succinate synthases and Mo-cofactor containing dehydrogenases. A review by T. Lueders focusing on BTEX biodegradation summarizes the microorganisms and microbial interactions involved in the process (Lueders, 2017). Nevertheless, to develop effective bioremediation technologies using such microorganisms, there is a need for a better insight into the molecular mechanisms/events on both cell and community level. Exploring the composition and critical players of the microbial community is essential for their applications in the bioremediation technologies (Kleinsteuber et al., 2012; Bacosa et al., 2018). Considering the environmental parameters and available microorganisms (native or non-native), Ławniczak and colleagues reviewed the current hydrocarbon bioremediation strategies (Ławniczak et al., 2020).

With the help of genomics, transcriptomics and proteomics, a deeper understanding of hydrocarbon biodegradation in pure cultures can be achieved. Additionally, metabolomics can elucidate the metabolites present during hydrocarbon biodegradation in pure cultures or consortia. However, culture-dependent methods have severe limitations when it comes to the investigation of microbial communities. Most of the microorganisms are difficult or even impossible to culture under laboratory conditions. Thus, their functions cannot be fully resolved by classical, standard approaches. The constant development of high throughput data generation and assessment has opened new frontiers in the research of hydrocarbon biodegradation. For examination of entire hydrocarbon-degrading communities, “meta-omics” approaches, including metagenomics, metatranscriptomics, metaproteomics, have been developed.

In the next chapters, the current knowledge about anaerobic hydrocarbon-degrading microorganisms, metabolic pathways, microbial interactions in the multi-omics era is overviewed.

## Multi-Omics Techniques Applied in Anaerobic Hydrocarbon Biodegradation Research

The classical culture-based microbiological, molecular biological and biochemical methods have several limitations, including the problems regarding the unculturable microbes or studying a single microbe or a community at the system level. Powerful techniques of the multi-omics era overcome the problems of conventional biochemical and microbiological methods to provide a system-wide picture of the capabilities, molecular mechanisms and interactions of the cells and consortia during hydrocarbon mineralization. Most of the pollutants are composed of various hydrocarbons; their bioconversions require various metabolic pathways usually present in distinct microbes. Therefore, complex contaminations can only be eliminated by the concerted action of diverse microbial communities. Understanding such complicated systems needs high throughput approaches enabling to study the processes at the system level. In this chapter, we present a brief overview of the widespread branches of the omics. Table 1 summarizes recent studies on anaerobic hydrocarbon biodegradation utilizing multi-omics techniques.

**TABLE 1:** Recent studies using omics approaches to characterize anaerobic hydrocarbon biodegrading microbial communities.

Omics approaches	Major findings	Citation
Genomics	Novel genomic features associated with the energy metabolism of *Geobacter ferrireducens* IRF9 strain.	Jung et al., 2018
Proteomics and gene expression study (by qPCR)	The regulation of benzylsuccinate synthase expression in *Magnetospirillum sp.* strain 15-1 has an extra layer on the post-transcriptional level to better cope with the redox dynamics of the environment. Various sensory inputs play an important role in regulation.	Meyer-Cifuentes et al., 2020
16S rDNA profiling and whole metagenome sequencing and metabolomics	Enrichment cultures derived from seafloor sediments are capable of anaerobic degradation of hexadecane and phenanthrene. Sulfate-reducing bacteria were identified as the main actors of the microbial community, along with syntrophic partners.	Shin et al., 2019
16S rDNA profiling	An anaerobic phenanthrene degrading consortium was successfully used for producing electricity in a microbial fuel cell.	Sharma et al., 2020
16S rDNA profiling and whole metagenomics was combined with qPCR studies	Novel fumarate adding enzyme was discovered from metagenomic data which is hypothesized participating in o-xylene activation.	Rossmassler et al., 2019
16S rDNA profiling	Four keystone microbial taxa SAR202 clade, *Thermoanaerobaculum*, *Nitrospira*, and *Xanthomonadales* were identified in PAH-contaminated soil samples with co-occurrence analysis.	Geng et al., 2020
16S rDNA and *bamA* gene profiling	*Anaerolineaceae*, *Dechloromonas*, *Bacteroidetes* Vadin HA17 and *Geobacter* were found as keystone microorganisms in PAH biodegradation under nitrate-reducing conditions.	Han et al., 2021
Whole metagenomics	The presence of methane metabolism and sulfur reduction genes were detected in Korarchaeota. Methane metabolism was suggested as an early energy conservation strategy in Archaea. Furthermore, the authors proposed the new archaeal species *Methanodesulfokores washburnensis*.	McKay et al., 2019
16S profile, whole metagenomics, comparative genomics, metatranscriptomics	Manganese reducing *Candidatus Methanoperedens spp.* are capable of reverse methanogenesis.	Leu et al., 2020
Reanalysis of metagenomes	Putative multi-carbon oxidizing *mcr* genes were found in many archaeal phyla.	Wang et al., 2019
Whole metagenome analysis	Horizontal gene transfer of *mcr* genes into Asgard group archaea was demonstrated. The oxidation of short-chain alkanes via alkyl-CoM was also suggested.	Seitz et al., 2019
Whole metagenomics combined with metabolomics	Acetate and hydrogen are the central metabolites on which the biochemical interactions between community members rely in deep-sea sediments. Acetate consumption is strongly connected to sulfate reduction, organohalide respiration and acetoclastic methanogenesis while hydrogen consumption can promote carbon fixation. Upstream actors of the biochemical pathways will degrade necromass and hydrocarbon compounds in the sediment on an acetogenic and hydrogenogenic manner.	Dong et al., 2019
Whole metagenome sequencing	The full sett of fumarate addition genes was found in the members of the candidate phylum Atribacteria. The metabolic capabilities of the phylum members suggest their importance in the carbon cycle of hydrocarbon-rich environments.	Liu et al., 2019
Whole metagenome sequencing	Assimilatory sulfate and dissimilatory nitrate reduction are dominant in a petroleum-contaminated aquifer. While the environment is anaerobic, many genes were found in the metagenomic data corresponding to aerobic biodegradation.	Cai et al., 2019
16S rDNA profile and metatranscriptomics	RNA seq data revealed that a group of nitrifiers insignificant in number has a rather significant impact on the metabolic pathways by supplying nitrate to the nitrate reducers in activated sludge bioreactors.	Sato et al., 2019
Metatranscriptomics	Nitrate and sulfate metabolism is connected to aerobic hydrocarbon biodegradation and methane production/oxidation in freshwater sediments.	Reid et al., 2018
Metatranscriptomics	Versatile metabolic pathways were found active in the hydrocarbon contaminated Detroit River sediment. Transcriptomics data showed that aerobic hydrocarbon biodegradation was closely connected to nitrate reduction, acetogenesis, methanogenesis, polyester synthesis and gluconeogenesis.	Falk et al., 2019
Metatranscriptomics	A nitrate-reducing consortium degrades benzene mainly via carboxylation. The involvement of a facultative anaerobe pathway during downstream bioconversion of benzene and the evolution of oxygen from nitrate was also suggested.	Atashgahi et al., 2018
Whole metagenomics and metaproteomics	A metabolic model for *G. metallireducens* which suggests that the catabolic pathways for the favored substrates -like toluene- are expressed continuously even if the substrate is not present.	Marozava et al., 2020
16S rDNA profile, whole metagenomics, metabolomics	Metagenomic data showed that sulfate-reducing bacteria responsible for oil pipe corrosion are able to thrive in the pipes despite nitrate treatment.	Bonifay et al., 2017

### Genomics, Transcriptomics and Proteomics on Pure Cultures

After isolating a hydrocarbon-degrading microorganism, its metabolic potential is investigated via whole-genome sequencing (WGS). The assembled genomes provide a blueprint of the organism and all the biochemical pathways it possesses. Moreover, knowledge of the full genome sequence makes taxonomic identification easier and the databases built from the genomes serve as templates in the metagenomic databases, analysis. Many genomes of anaerobic hydrocarbon biodegraders have been published (Rabus et al., 2005; Mattes et al., 2008; Aklujkar et al., 2009; Selesi et al., 2010; Jiang et al., 2012; Martín-Moldes et al., 2015; Yin et al., 2017). Comparative analysis of the genome of the iron-reducing bacterium *Geosporobacter ferrireducens* IRF9 identified multiple anaerobic hydrocarbon activating genes (alkylsuccinate synthase) harbored by the strain (Jung et al., 2018).

The genome of an organism harbors vast numbers of enzymes for many metabolic pathways, but specific the DNA sequence itself does not reveal their activity under certain conditions or in the presence of specific carbon sources. Transcriptomic and proteomic analysis shed light on the active pathways during hydrocarbon biodegradation. Numerous studies have been published on whole-cell transcriptomic responses of pure aerobic cultures to the presence of hydrocarbons and other xenobiotics (Sabirova et al., 2006, 2011; Laczi et al., 2015; Hegedüs et al., 2018). An early study of the marine sulfate-reducing strain NaphS2 revealed the key enzymes of anaerobic naphthalene biodegradation with the combined power of draft genome sequencing, RNA microarray and proteomics (Di Donato et al., 2010). In a proteomics study, Ralf Rabus and colleagues elucidated the catabolic network of *Aromatoleum aromaticum* EbN1 during aromatics biodegradation applying more than 50 growth conditions. They identified 20 new proteins participating in the peripheral pathway of anaerobic aromatics mineralization and tested the effect of environmental conditions such as carbon limitation or solvent toxicity on the proteome of *A. aromaticum* EbN1 (Rabus et al., 2014). Microarray analysis and qPCR experiments shed light on the transcriptional regulation of gene products involved in alkylsuccinate metabolism in *Desulfatibacillum alkenivorans* AK-01 (Herath et al., 2016). Gene expression experiments combined with proteomics recently provided evidence on the post-translational regulation of benzylsuccinate synthase in *Magnetospirillum* sp. 15-1 (Meyer-Cifuentes et al., 2020). However, combined studies of transcriptomics and proteomics are still rare. Transcriptomic data, as the final output of gene expression, must be handled carefully, since mRNA levels and protein levels are not always proportional. Buccitelli and Selbach argue in their recent review that integrated transcriptomics and proteomics should become a common integrated approach and quantifying protein and mRNA levels (which are the result of synthesis and degradation) would provide a complete picture on gene expression dynamics (Buccitelli and Selbach, 2020).

Although studying individual isolates is an important way to reveal molecular mechanisms behind anaerobic hydrocarbon bioconversion, usually the microorganisms do not act alone in the environment. Therefore, the anaerobic biodegradation of hydrocarbons is mainly studied in microbial communities. We discuss further examples and techniques in the following chapters.

### Metagenomics

A cost-effective technique to elucidate the composition and functional capabilities of a hydrocarbon-degrading microbial community is metagenomics. There are two major approaches (1) amplicon sequencing (or targeted metagenome sequencing) and (2) whole metagenome sequencing (mWGS) or shotgun metagenome sequencing. In the case of targeted metagenome sequencing, specific genes (targets) are amplified by PCR then sequenced on second- or third-generation sequencing platforms. The most common target is the V3–V4 hypervariable region of the 16S rRNA gene, but other regions, including V1–V2, V6–V8 or even the whole sequences (Johnson et al., 2019), are also used. The choice of the region can have a significant effect on taxonomic resolution (Graspeuntner et al., 2018; Bukin et al., 2019; Johnson et al., 2019). With a 16S rDNA gene profile, we can get a picture of the taxonomic composition of hydrocarbon-degrading bacterial communities. Besides the taxonomic classification, the richness and diversity of the samples can also be estimated (Potts et al., 2019). Co-occurrence of microorganisms in multiple hydrocarbon-contaminated samples suggests their association and microbial networks can be built from co-occurrence studies (Geng et al., 2020; Tikariha and Purohit, 2020; Han et al., 2021).

Genes of selected function are also utilized in targeted metagenomics, which narrows the analysis to particular group of microbes and/or a function in the community. Such genes can be, for example, *mcrA* for methanogenic archaea (Denonfoux et al., 2013), the catalytic domain of alkyl- and benzylsuccinate synthases (*assA*/*masD*, *bssA*) and the 6-oxocyclohex-1-ene-1-carbonyl-coenzyme A hydrolases (*bamA*) of anaerobic hydrocarbon degraders (Kuntze et al., 2008; Stagars et al., 2016; Sperfeld et al., 2018). Using functional genes in amplicon sequencing, one can predict the hydrocarbon-degrading potential of a microbial community.

For evaluation of 16S and other target gene profiles, there are many bioinformatical tools. Table 2 reports the most frequently used programs such as BLAST or MALT combined with Megan (Huson et al., 2007, 2016). Qiime 2, for example, is a recently developed pipeline (Hall and Beiko, 2018; Bolyen et al., 2019). Because it is robust, Qiime 2, and its predecessor Qiime, are widely used in 16S profile analysis of hydrocarbon-degrading microbiomes (Mejeha et al., 2019; Shin et al., 2019; BenIsrael et al., 2020; Sharma et al., 2020) and other microbial communities.

**TABLE 2:** Bioinformatic tools used in the multi-omics analysis.

Name	Application	Databases	Citation
Megan	A multitool for taxonomic and functional analysis of metagenomes and amplicon profiles with a graphical user interface and options for PCoA and cluster analysis.	NCBI nr protein for eggNOG, InterPro2GO and SEED, NCBI nucleotide database, Silva SSU and LSU rRNA databases, KEGG (for Ultimate edition only).	Huson et al., 2007, 2016
Qiime 2	Integrating multiple algorithms for analyzing amplicon sequencing data from quality control to taxonomic identification. Also suitable for analyzing diversity and richness.	Silva SSU and LSU rRNA databases, Greengenes 16S rRNA database, Unite ITS database, custom made databases are also supported.	Hall and Beiko, 2018; Bolyen et al., 2019
Dada2	An open-source R package for modeling and correcting Illumina amplicon sequencing errors. It is also suitable for taxonomy assignment. Output data can be imported into Phyloseq for further analysis. It is also implemented in Qiime2.	RDP, Greengenes, Silva SSU and LSU rRNA databases, Unite (for fungal taxonomy), custom made databases are also supported.	Callahan et al., 2016
Phyloseq	An open-source R package for a variety of analysis like diversity multi-table comparison etc.	none	McMurdie and Holmes, 2013
Vsearch	A versatile open-source tool for the evaluation of amplicon sequencing also implemented into Qiime2.	Uchim “Gold” database (for chimera filtering if not *de novo* chimera search is used), Greengenes, Silva SSU and LSU rRNA databases, Unite (for fungal taxonomy), custom made databases are also supported.	Rognes et al., 2016
Diamond	A fast sequence aligner for protein and translated DNA sequences working up to 20000 times faster than BLAST. Can be used as stand-alone but the output is compatible with other softwares, such as Megan.	NCBI nr or other protein databases. Custom made databases are also supported.	Buchfink et al., 2014
Kaiju	A fast k-mer based sequence taxonomy assignment for reads of whole metagenomics or metatranscriptomics data.	There is a number of protein databases prebuilt from the NCBI, Mar and RVB-prot. Costume databases are also supported.	Menzel et al., 2016
Kraken 2	K-mer based taxonomic sequence classifier.	A number of protein and nucleotide databases are available like RefSeq, NCBI nr and nt, UniVec_Core etc…	Wood et al., 2019
SPAdes/metaSPAdes	MetaSPAdes is designed for assembling shotgun metagenomic reads. It is relying on Spades.	none	Nurk et al., 2017
Idba-UD	Open source de Bruijn graph assembler for single-cell and metagenomic sequencing data.	none	Peng et al., 2012
MEGAHIT	Open source ultra-fast assembler optimized for metagenomics data.	none	Li et al., 2015
Metabat 2	Adaptive binning algorithm using tetranucleotide frequency and abundance scores for recovering MAGs from Metagenomic data.	none	Kang et al., 2015, 2019
MaxBin 2	Binning software for recovering MAGs.	none	Wu et al., 2015
Concoct	Binning software for recovering MAGs.	none	Alneberg et al., 2014
Metawrap	A pack of tools for assembling metagenomes, binning, refining and annotating MAGs.	NCBI nr database	Uritskiy et al., 2018
DAS Tool	A bin refinement software.	none	Sieber et al., 2018
checkM	A pack of tools for assessing the quality of genomes and MAGs.	Own database with high-quality genomes to establish the marker genes for bin identification.	Parks et al., 2015
MAGpy	A pipeline for downstream analysis of MAGs.	Uniprot, Sourmash, Pfam, checkM.	Stewart et al., 2018
PhylophlAn	A pipeline for large-scale phylogenetic profiling of genomes and metagenomes.	Marker gene databases: PhyloPhlAn, AMPHORA2.	Asnicar et al., 2020
Galaxy	A web-based tool collection for multiple omics analysis.	Multiple databases.	Afgan et al., 2018
MG-RAST	Web-based service for microbiome studies.	Multiple databases.	Keegan et al., 2016
MGnify	A web-based pipeline by EBI for microbiome studies.	Multiple databases.	Mitchell et al., 2020
OneCodex	A fast web-based pipeline for microbiome studies contains free and paid services.	RefSeq and OneCodex databases.	Minot et al., 2015

If we want to inspect the overall metabolic potential of a hydrocarbon-degrading community, we need to apply the mWGS method. Shotgun sequencing data can be processed in two ways (1) read-based analysis and (2) assembly based analysis.

In the first case, the reads are utilized to obtain taxonomic and functional information. Reads are aligned with the NCBI RefSeq or the NCBI nr protein database. Diversity also can be calculated from the read count. In the second case, the reads are assembled into contigs with an appropriate assembler (Ayling et al., 2019), and individual genomes are recovered with binning methods (Sangwan et al., 2016; Sieber et al., 2018; Uritskiy et al., 2018). The quality of the bins is characterized by completeness and contamination (Parks et al., 2015).

Metagenomic approaches are the most frequently used techniques among the “omics” for investigation of - hydrocarbon-degrading - microbial communities. In the first half of the last decade, many microbiomes were studied in various anaerobic hydrocarbon-contaminated niches, including crude oil (An et al., 2013b; Hu et al., 2016; Nie et al., 2016) and coal reservoirs (An et al., 2013b; Lawson et al., 2015), hydrothermal vents (He et al., 2013, 2015; Oulas et al., 2016), natural oil seeps (Håvelsrud et al., 2011; Hawley et al., 2013, 2014), hydrocarbon-contaminated terrestrial (Abbai and Pillay, 2013) and marine environments (Kimes et al., 2014). Metagenomic data can provide valuable information on the metabolic capabilities of known and newly discovered, culturable and non-culturable microorganisms as well. Some examples are discussed here from recent years. Metagenome-based evidence on the utilization of hydrocarbons via fumarate addition in *Smithella sp.* and members of the *Anaerolineaceae* family has been published (Tan et al., 2014; Rossmassler et al., 2019). There is a vast number of uncultured bacterial species in hydrocarbon-associated deep-sea sediments. The members of two candidate phyla, *Ca.* Atribacteria and *Ca.* Bathyarchaeota, were found predominantly in the hydrocarbon-containing sediments of the Gulf of Mexico. 13 metagenome-assembled genomes (MAGs) (*Chloroflexi*, *Aminicenantes*, *Aerophobetes*, *Actinobacteria*, *Ca. Bathyarchaeota*, *Thorarchaeota*, and *Lokiarchaeota*) harbored glycyl-radical enzymes catalyzing the fumarate addition pathway (Dong et al., 2019). In another recent study, MAGs and SAGs (single-cell amplified genomes) were reanalyzed and linked anaerobic hydrocarbon biodegradation by fumarate addition to *Ca. Atribacteria*. The authors identified four novel *Atribacterial* lineages and analyzed their metabolic capabilities (Liu et al., 2019). In a petroleum-contaminated aquifer, nitrate and sulfate reducers dominated the community. Although the aquifer was anaerobic, multiple aerobic biodegradation genes were found in the samples, but genes of enzymes involved in methanogenesis were lacking (Cai et al., 2019). In certain environments having fluctuating levels of dissolved oxygen, both anaerobic and aerobic metabolic pathways could be detected in the metagenomes. For example, the co-occurrence of aerobic and anaerobic aromatics degraders in oil-impacted mangrove sediments was recently reported (Sousa et al., 2020).

### Metatranscriptomics

Shotgun metagenome sequencing provides an overwhelming amount of information on the composition and potential metabolic pathways existing in a microbiome. On the other hand, it fails to fully resolve the active members, components and pathways of the community and distinguish them from the inactive ones. The taxonomic profile will elucidate the most active community members, while assembly of transcriptomic data can provide information on the active pathways at the cell or community level (Shakya et al., 2019). In a recent study, Sato and colleagues showed that, contrary to their low abundance, nitrifiers play an important role in heavy oil biodegradation by providing nitrate to denitrifying hydrocarbon degraders in activated sludge reactors (Sato et al., 2019). Another study showed both nitrogen and sulfur cycles are active alongside methanogenesis and methane oxidation in hydrocarbon-containing freshwater sediments (Reid et al., 2018). Falk and colleagues reported that nitrate reduction and methanogenesis are the most abundant pathways in the hydrocarbon-contaminated Detroit River (Falk et al., 2019). Another metatranscriptomic study suggests the co-occurrence of aerobic and anaerobic benzene degradation pathways, which are enabled by nitrate/nitrite reduction driven oxygen evolution catalyzed by a nitric oxide dismutase (NOD) (Atashgahi et al., 2018).

### Metaproteomics

The metaproteome is the expressed protein complement of a microbiome (Rodríguez-Valera, 2004; Wilmes and Bond, 2004; Pieper et al., 2013). Since proteins provide biological functions for an organism, metaproteomics shed light on the metabolic activity and dynamics of a microbial community at the protein level (Pieper et al., 2013). Besides, protein-based stable isotope probing (protein-SIP) of a metaproteome yields information on protein activity and turnover (Jehmlich et al., 2016), while stable isotope fingerprinting (SIF) will elucidate carbon sources and assimilation pathways used by the community members (Kleiner et al., 2018). Moreover, a method based on protein abundance has been recently developed for accurate assessment of the microbial community structure and species biomass contributions (Kleiner et al., 2017). With the development of tandem LC-MS/MS methods, new opportunities have opened for fast and accurate proteomic analysis. In the last decade, 2D gel electrophoresis has been replaced by more rapid online liquid chromatography separation. A recently published paper evaluates multiple online and offline LC-MS/MS pipelines and provides a decision chart for the most appropriate choice of method for any budget and purpose (Hinzke et al., 2019). Although metaproteomics is a powerful method to study the biochemical functions of microbial communities, it is in its infancy and has some limitations (Abiraami et al., 2020). Therefore, only a few studies used metaproteomics to examine anaerobic hydrocarbon biodegradation.

Benndorf and colleagues investigated the efficiency of metaproteomics on anaerobic benzene degrading communities (Benndorf et al., 2009). They concluded that metaproteomics is a useful method to examine unculturable microbes; however, the protein extraction methods should be adapted to the slow-growing anaerobic microbial communities. Bargiela and colleagues combined metaproteomics and metabolomics in a study of three chronically polluted sites in the Mediterranean sea. Their results revealed the prevalence of C_1_-compound metabolism. Methane oxidation was detected on the oxygen-rich site while methanol catabolism was observed on all polluted sites (Bargiela et al., 2015). In a recent study, Marozava and colleagues used metagenomics and metaproteomics to establish a specific expression profile of catabolic pathways for aromatics biodegradation by *Geobacter metallireducens* during sessile growth (Marozava et al., 2020).

### Metabolomics

The collection of all biochemical molecules, substrates, products, intermediates existing in a living organism is called the metabolome. Similarly to the transcriptome and proteome, it is dynamic in time; moreover the metabolome might be considered as the most sensitive, most dynamic system in the cells. Metabolome, the result of cell metabolism, serves as the “ultimate proof” for biochemical reactions taking place in living systems (Liu and Locasale, 2017).

Metabolome research can be divided into three parts: non-diffusible, diffusible and epi-metabolome. The non-diffusible metabolome will never leave the cell, while the diffusible metabolome can escape the cell occasionally depending on the metabolic rate of the cell. From the three parts, the epi-metabolome has the lowest conversion rate so it can diffuse out, or be actively transported into the environment then captured and further metabolized by other members of the community. The epi-metabolome is the only part of the metabolome which moves freely at the contaminated site. Molecules belonging to the epi-metabolome are metabolized by the microbial network (De Lorenzo, 2008). The redox electron carriers of the epi-metabolome have outstanding importance in the case of exo-electrogenic microbes (see below).

In anaerobic hydrocarbon biodegradation studies, metabolomics is usually used to search for signature molecules of hydrocarbon degradation pathways. The hydrocarbon substituted succinate derivatives are excellent candidates for signature molecules since they are unique to anaerobic hydrocarbon biodegradation (Bian et al., 2014, 2015; Chen et al., 2020). Metabolomic studies are often combined with metagenomic approaches. In a recent study, Dong and colleagues investigated oil seepage in deep-sea sediments and concluded that acetate and hydrogen are central intermediates, supporting metabolic interactions between community members (Dong et al., 2019). Oil companies often use nitrate as an additive to fight off sulfate-reducing bacteria, thus preventing crude oil souring and corrosion of transport and storage vessels. Bonifay and colleagues investigated the corrosion of oil production pipelines with the help of metagenomics combined with metabolomics. They found that the addition of nitrate is not enough to prevent oil pipeline corrosion because sulfate reducers can survive in biofilms, thus evading the effect of nitrate and facilitating oil souring and pipe corrosion (Bonifay et al., 2017).

## Metabolic Aspects of Hydrocarbon Biodegradation

Microbial biodegradation of hydrocarbons starts with an activation reaction, both aerobically and anaerobically. In aerobic pathways, mono- and dioxygenases catalyze this reaction by utilizing molecular oxygen to synthesize the corresponding alcohol from the hydrocarbon. On the other hand, anaerobic activation of hydrocarbons can happen through several distinct reactions: fumarate addition, oxygen-independent hydroxylation, direct carboxylation, hydration and reverse methanogenesis.

### Aerobic Biodegradation of Hydrocarbons

Although the primary topic of this review is the anaerobic biodegradation of hydrocarbons, the importance of hydrocarbon utilization under aerobic conditions cannot be ignored. Thus, in the following paragraphs, a brief summary of aerobic hydrocarbon oxidation pathways and enzymes is given.

The initial reaction of aerobic alkane biodegradation is catalyzed by various monooxygenases (also known as alkane hydroxylases) depending on the chain lengths of the alkanes (Van Beilen and Funhoff, 2007; Wang and Shao, 2013). These include methane monooxygenases [either membrane-bound (pMMO) or soluble (sMMO)], propane and butane monooxygenases (Van Beilen and Funhoff, 2007), the CYP153 family of Cytochrome P450 enzymes (Funhoff et al., 2006) and alkane-1-monooxygenases (AlkB).

The biodegradation of aromatics can be divided into two subsequent pathways (Fuchs et al., 2011). In the peripheral pathway, the aromatic ring is converted into catechol or protocatechuate by ring-hydroxylating dioxygenases or monooxygenases. In the central pathway, the ring is opened by ring-cleaving dioxygenases in ortho meta or para positions. Then the catechol/protocatechuate is transformed into acetaldehyde/pyruvate or into β-ketoadipate, which is further converted into succinate and Ac-CoA.

### Anaerobic Biodegradation of Hydrocarbons

**FIGURE 1:**
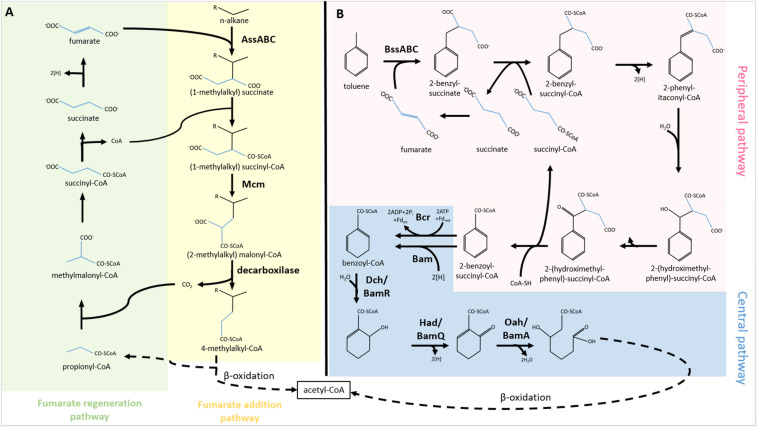
Activation of **(A)** alkanes and **(B)** aromatics through fumarate addition and their biodegradation under anaerobic conditions. AssABC, alkylsuccinate synthase; Mcm, methylmalonyl-CoA mutase; BssABC, benzyl-succinate synthase; Bcr/Bam, benzoyl-CoA reductase; Dch/BamR, dienoyl-CoA hydratase; Had/BamQ, hydroxyacyl-CoA dehydrogenase; Oah/BamA, oxoacyl-CoA hydrolase; Fd_ox_, oxidized ferredoxin; Fd_red_, reduced ferredoxin.

The most common and best-described anaerobic alkane activation reaction is fumarate addition (Figure 1A). Fumarate is fused with the alkane chain at the second carbon atom, forming a (1-methylalkyl)-succinate. The product is attached to an acetyl-coenzyme A and converted into (2-methylalkyl)-malonyl-CoA through a carbon skeleton rearrangement. Later 4-methylalkyl-CoA is formed and utilized in β-oxidation. In the first two cycles of β-oxidation, acetate and propionate are cleaved off the molecule. The propionate is recycled in the methylmalonyl-CoA pathway to form fumarate, which can be reutilized in (1-methylalkyl)-succinate synthesis (Davidova et al., 2005). The key enzyme of the fumarate addition reaction is identified as a glycyl radical enzyme named as alkyl succinate synthase or (methylalkyl)-succinase (Ass/Mas). The enzyme has three different subunits (AssABC). Mas genes were first described in the denitrifying *Aromatoleum* sp. strain HxN1 (Grundmann et al., 2008). Two genes (*assA1* and *assA2*), coding for the catalytic subunit of Ass, were found in various loci in the genome of *D. alkenivorans* AK-01 (Callaghan et al., 2012). Metagenomic analysis revealed that *Smithella sp.*, and *Anaerolinea sp.* also harbor *ass* genes (Tan et al., 2014; Rossmassler et al., 2019). In a recent study, *ass* and *bss* genes were detected from MAGs of the Asgard archaea (Farag et al., 2020).

Methanotrophic archaea can oxidize methane under anoxic conditions via reverse methanogenesis. The mechanism was predicted by Hallam and colleagues based on genomic data (Hallam et al., 2004). They found the gene of methyl-CoM reductase (Mcr) in the genome of methanotrophic archaea, which catalyze the final reaction of methanogenesis. The enzyme reduces methyl-CoM, resulting in methane and a heterodisulphide composed of coenzyme M and coenzyme B. Under suitable conditions, the process can be reversed by Mcr to form methyl-CoM. This hypothesis was proven by Scheller and colleagues (Scheller et al., 2010). Metagenomic analysis of a hot spring shed light on the coupling of methane and dissimilatory sulfur metabolism in Korarchaeota (McKay et al., 2019). A combined metagenomic and metatranscriptomic study showed that *Candidatus Methanoperedens* spp. is capable of reverse methanogenesis during manganese reduction (Leu et al., 2020).

Additionally, an alternative pathway for butane activation by Mcr proteins in thermophilic archaea has been proposed from a metagenomic analysis (Laso-Pérez et al., 2016), indicating possible activation pathways of non-methane alkanes. Wang and colleagues reanalyzed 64 metagenomes and found putative multi-carbon oxidizing *mcr* genes among many archaeal phyla (Wang et al., 2019). Horizontal gene transfer of *mcr* genes into the Asgard archaea was justified by metagenome analysis. The authors also suggested the oxidation of short-chain alkanes via alkyl-CoM in Helarchaeota (Seitz et al., 2019).

Two alternative activation pathways of alkanes were proposed in *Desulfococcus oleovorans*. In the first scenario, the carbon chain is carboxylated at the third carbon atom, forming a 1-ethyl alkanoate, which is further converted into acetate in β-oxidation (Meyer et al., 2003). In the other scenario, a subterminal hydroxylation occurs at C3, which is transformed into a branched fatty acid through a ketone intermediate. Genomic data support the latter since an ethylbenzene dehydrogenase (EBDH)-like enzyme is encoded in the genome of *D. oleovorans* (Rabus et al., 2016).

Similarly to aerobic biodegradation, utilization of aromatic compounds under anoxic conditions can be split into peripheral and central pathways (Fuchs et al., 2011; Figure 1B). In the peripheral pathways, the activation of the aromatic compound takes place through distinct enzymatic reactions. Under anoxic conditions, the aromatic molecules are converted into the central metabolite, benzoyl-CoA or its derivatives through several steps. First, fumarate is added to the aromatic compound by the glycyl radical enzyme, benzylsuccinate synthase (Bss). The enzyme occurred in several denitrifying bacteria, e.g., *Azoarcus* sp. (Achong et al., 2001) recently renamed to *Aromatoleum* sp. (Rabus et al., 2019) and *Thauera aromatica* (Leuthner and Heider, 2000). The enzyme is encoded by the *bssABC* genes forming an operon (Leuthner et al., 1998; Hermuth et al., 2002). Bss protein is one of the few enzymes involved in anaerobic hydrocarbon activation of which 3D structure has been determined. Based on X-ray diffraction study, the structure of Bss is a heterohexamer (αβγ)2 (Funk et al., 2014). The small subunits (β and γ) comprise 4Fe-4S clusters coordinated by four cysteines, whilst the large subunit (α) harbors the catalytic site (Funk et al., 2014). Another gene codes for a glycyl radical enzyme-activating protein, BssD (Leuthner et al., 1998; Hermuth et al., 2002). This *S*-adenosyl-methionine-dependent protein plays a role in the Bss precursor activation (Hermuth et al., 2002). The benzylsuccinate formed in the reaction is further converted into the central metabolite, benzoyl-CoA, by the enzymes encoded in the *bbs* operon (Leuthner and Heider, 2000).

Furthermore, the activation of polyaromatic hydrocarbons via fumarate addition has been suggested (Annweiler et al., 2000). Another glycyl radical enzyme, naphtyl-2-methylsuccinate synthase (Nms), was identified in a sulfate-reducing enrichment culture (Selesi et al., 2010). Nms is closely related to Bss; however, they are clustered into two separate groups on the phylogenetic tree (Von Netzer et al., 2013; Heider et al., 2016).

On the other hand, the activation of aromatics can be performed through oxygen-independent hydroxylation. The mechanism was studied on ethyl- and propylbenzene. A molybdenum/iron-sulfur/heme-enzyme, ethylbenzene dehydrogenase (EBDH), catalyses the reaction in the periplasm of *A. aromaticum* EbN1 (Kniemeyer and Heider, 2001). The enzyme consists of three subunits, two of which (αβ) have five iron-sulfur clusters together. The molybdenum-BisMGD cofactor can be found in the α subunit, along with one of the five iron-sulfur clusters (FS0). The γ subunit harbors the heme b cofactor (Kloer et al., 2006). The enzyme can convert a broad range of substrates (Szaleniec et al., 2007; Knack et al., 2012) and has a higher binding affinity toward hydrophobic compounds; nevertheless, substrates with electron-donating substituents in the para position can elevate the conversion rate (Knack et al., 2012). EBDH and similar enzymes, as well as their roles in oxygen-independent hydroxylation, have been reviewed (Heider et al., 2016).

Direct carboxylation of benzene has also been reported in a few studies (Musat and Widdel, 2007; Kunapuli et al., 2008; Abu Laban et al., 2009). However, benzoic acid, which was detected as an intermediate, is also the central metabolite in the anaerobic biodegradation of aromatic compounds (Fuchs et al., 2011); therefore, other activation mechanisms could not be excluded. The proteomic study of an iron-reducing enrichment culture revealed the benzene-dependent induction of carboxylase-like proteins (Abu Laban et al., 2010). Similarly, in a metatranscriptomic study of a nitrate-reducing enrichment, the authors found benzene-induced expression of carboxylases (Luo et al., 2014). These findings support the hypothesis of direct carboxylation as an alternative activation reaction, although no direct evidence for the existence of this mechanism was reported.

There are two different routes for the central pathway for anaerobic biodegradation of aromatics (Figure 1B). One is ATP dependent, whilst the other is ATP independent. The ATP-dependent pathway begins with a reduction reaction catalyzed by a class I benzoyl-CoA reductase (Bcr) (Boll and Fuchs, 1995). The four subunits of the heterotetrameric enzyme are encoded by the *bcrABCD* genes, which are organized into an operon in *Thauera aromatica*. The enzyme cleaves ATP into ADP + P_i_ during the reduction process and accepts ferredoxin as an electron donor. The presence of an ATP-dependent benzoyl-CoA reductase in the hyperthermophilic archaeon *Ferroglobus placidus* was recently demonstrated (Schmid et al., 2016). Apart from the central dearomatisation reaction, the role of Bcr in dehalogenation of chlorinated, fluorinated and brominated aromatics was shown (Tiedt et al., 2016).

The ATP-independent pathway is catalyzed by a class II benzoyl-CoA reductase (Bam). The function of the Bam protein was first suggested by Laempe and colleagues (Laempe et al., 1999). This enzyme class is found in strictly anaerobic bacteria, including the iron reducer *G. metallireducens* (Wischgoll et al., 2005). The enzyme is composed of eight subunits which are encoded by *bamBCDEFGHI* genes in one operon with *bamA* which is a hydrolase and catalyses the final ring cleavage in the ATP-independent pathway (Löffler et al., 2011). A differential membrane proteome analysis of *G. metallireducens* revealed the association of Bam protein complex to the membrane (Heintz et al., 2009). The Bam enzyme was identified distinctly in iron-reducing (Wischgoll et al., 2005) and sulfate-reducing bacteria (Kim et al., 2014; Dong et al., 2016). The structure and function of Bam have been reviewed (Boll et al., 2016).

## Syntrophy in Hydrocarbon-Degrading Microbial Communities

The compositions of microbial communities present in hydrocarbon-contaminated sites are highly dependent on the composition of the pollutants, physico-chemical properties of the environment and electron acceptors available. Electron acceptors, including nitrate, iron and sulfate, can be depleted relatively rapidly. Under these conditions, a syntrophy between hydrocarboclastic bacteria, methanogenic archaea and other microbial partners is established. Nevertheless, syntrophy is a crucial mechanism of anaerobic hydrocarbon biodegradation not just under metanogenic conditions but in the presence of sulfate, ferric ion or even nitrate (Kleinsteuber et al., 2012). Even though syntrophic communities are common under anaerobic hydrocarbon-degrading conditions, there is limited information as regards the interactions between community members (Gieg et al., 2014). With the emergence of new high-throughput sequencing techniques in the last decade, new perspectives have opened for the study of microbial communities. Metagenomic analysis of samples derived from hydrocarbon-contaminated environments highlighted the role of the δ-proteobacterial class *Syntrophaceae* in hydrocarbon biodegradation under methanogenic conditions (Siddique et al., 2011; An et al., 2013a; Embree et al., 2014; Wawrik et al., 2016). The role of *Peptococcaceae* in methanogenesis coupled to n-alkane biodegradation has also been endorsed (Abu Laban et al., 2015; Tan et al., 2015; Mohamad Shahimin et al., 2016; Mohamad Shahimin and Siddique, 2017a,b). Also, oil degraders belonging to other bacterial groups, for instance, members of *Chloroflexi*, like *Anaerolinea*, have been identified. These bacteria are thought to be scavenging dead cells and metabolites that are derived from hydrocarbon biodegradation (Gieg et al., 2014). The involvement of *Anaerolinea* in the primary degradation of hydrocarbons has been proposed (Liang et al., 2015, 2016; Mohamad Shahimin and Siddique, 2017a,b). Both acetoclastic and hydrogenotrophic methanogenic archaea are found in hydrocarbon-contaminated soil and sediments. Nonetheless, their ratio depends on the composition of the entire community (Siddique et al., 2011; Wawrik et al., 2016; Mohamad Shahimin and Siddique, 2017a).

**FIGURE 2:**
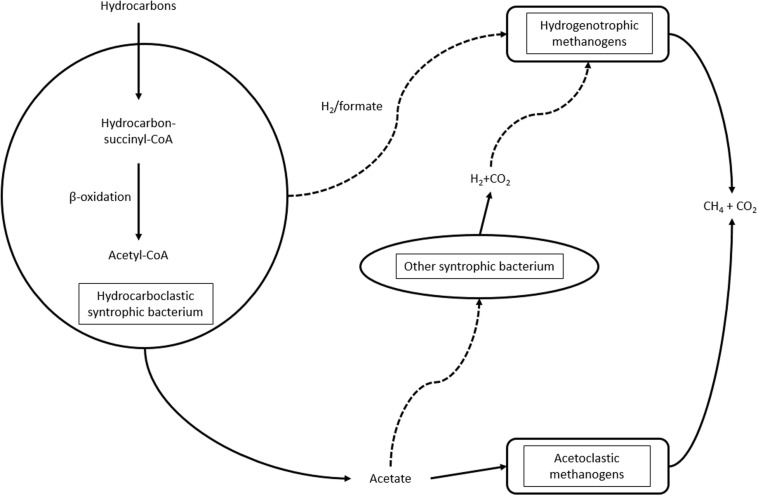
Syntrophic interactions during hydrocarbon biodegradation under methanogenic conditions.

The energy transfer between hydrocarbon degraders and methanogenic archaea is complex and not well-understood. According to the model (Embree et al., 2014; Gieg et al., 2014; Wawrik et al., 2016; Figure 2), the hydrocarbon degrader may provide electrons to methanogens through H_2_/formate. Hydrogen, along with carbon dioxide, is utilized by the hydrogenotrophic methanogenic archaea for methane evolution. Acetoclastic methanogens utilize acetate derived from hydrocarbon biodegradation. Additionally, other microorganisms, such as *Desulfuromonas sp.* (Wawrik et al., 2016), can also utilize acetate for hydrogen evolution, supplying the hydrogenotrophic methanogens. Hydrogenase and dehydrogenase enzymes are important participants in syntrophic communities (Wawrik et al., 2016), so a statistical method was developed to recognize communities with syntrophic potential from metagenomic data (Oberding and Gieg, 2016).

## Phytomicroremediation: Plant-Microbe, Microbe-Microbe Interactions

In the case of contaminants spread over a large area, the application of phyto-micro- bioremediation can be a cost and environmentally sound technology. Phytoremediation is a well-known technology in which microorganisms complement the role of plants. The interactions between the microbes and plants can be fruitful cooperation not only in the aerobic zone but the deeper level of soils and waters. Roots of plants provide nutrients and can improve the soil consistency that is especially beneficial in the deeper soil region. There are only a few species of plants with long roots able to achieve the 7–8 m depth (poplar, willow). *Spartina* species used in the remediation of oil-contaminated wetland enhanced the aerobic biodegradation by transferring oxygen to their roots (Lin and Mendelssohn, 1998).

Bioaugmentation of contaminated sites sometimes leads to unexpected modification in the rhizosphere. Recently published results justify that horizontal gene transfer occurred during rhizoremediation of organic compounds (Fernández et al., 2012; Segura and Ramos, 2013). In these cases, the microbe(s) used for biodegradation can not survive in the target environment, but the genes, metabolic routes of biodegradation are transferred into the local microbes; thus the bioconversion can take place. On the other hand, phytoremediation alone cannot be purely assigned to the metabolic activity of the plants; several studies linked the success of phytoremediation to the microbial activities of the rhizomicrobiome, but more likely it should be studied at metaorganism (host + microbiome) level (Thijs et al., 2016). Since the rhizosphere microbiome is sensitive to both pollutants and treatments, a competitive plant-growth-promoting and catabolic microbiome should be selected for efficient remediation and plant growth (Thijs et al., 2016). However, this makes the picture most complex since host-microbe and microbe-microbe interactions must be considered.

## Electron Transfer Between Bacterial Cells During Hydrocarbon Biodegradation

Methanogenic archaea live in syntrophy with bacteria that can break down complex organic compounds, such as plant parts or diesel oil, into simple molecules including acetate. Interspecies electron transfer (IET) is a critical element of syntrophic microbial communities. IET is a mechanism wherein the syntrophic partners share electrons through mediators (Mediated Interspecies Electron Transfer or MIET) or directly (Direct Interspecies Electron Transfer or DIET). Hydrogen functions as the dominant mediator in such communities, even though formate is energetically more favorable (Storck et al., 2016). Apart from this, quinone-mediated interspecies electron transfer (QUIET) was recently proposed for *G. metallireducens*/*G. sulfurreducens* co-cultures (Smith et al., 2015). Quinone moieties are abundant in many soil types as components of humic substances (Gralnick and Newman, 2007). In the case of QUIET, these quinones are utilized by the microbes as electron shuttles (Smith et al., 2015). In another study, the transition of electrons from the donor species to the acceptor species via conductive minerals has been reported (Kato et al., 2012). Recent studies disclosed that the DIET process might take place in co-cultures of various microorganisms in which *Geobacter* species play the leading role as primary degraders and electron donors for methanogens or other syntrophic partners (Rotaru et al., 2014a,b; Wang et al., 2016). Based on this knowledge, *Geobacter* strains appear to be potential vital partners in anaerobic bioremediation processes. Additionally, a recent study validated that DIET also works with cytochrome type C with no conductive pili between pili-deficient *Geobacter* species (Liu et al., 2018). The findings propose that DIET is more common and widespread among syntrophic communities than first presumed.

In exceptional cases, such as in seasonally anoxic sediments, the electron donor and the electron acceptor are spatially separated. Members of the *Desulfobulbaceae* family, however, can bridge the distance between the electron donor and acceptor with long-distance electron transfer (LDET) (Pfeffer et al., 2012; Bjerg et al., 2018). These so-called cable bacteria live in coastal marine or freshwater sediments. They form several centimeter-long filamentous multicellular structures with thousands of cells in them. The electron donor for the reaction is sulfide, which is oxidized by the cable bacteria several centimeters deep in the sediment. Electrons are then transferred via the suboxic zone, where both sulfide and oxygen are lacking. The terminal electron acceptor for cable bacteria is oxygen; however, they are also capable of nitrate respiration (Marzocchi et al., 2014; Seitaj et al., 2015). They have a thick-ridged envelope along the filament. It was suggested that the envelope plays a dual role as a brace and an electron conductor (Cornelissen et al., 2018). Although the exact mechanism of LDET is not well-understood, recent studies have indicated that cytochrome type C plays an essential role in it (Bjerg et al., 2018). Interactions between cable bacteria and other microorganisms have yet to be investigated; nonetheless, several models have been suggested. These models include MIET and DIET, where bacterial partners pass their electrons to cable bacteria (Meysman, 2018). By participating in the sulfur cycle, cable bacteria can facilitate the biodegradation of hydrocarbons and other pollutants by sulfate-reducing microorganisms. Despite the potential of cable bacteria in hydrocarbon removal, only a few studies were performed in this field (Müller et al., 2016; Matturro et al., 2017). This process can be enhanced by applying a bioelectrochemical snorkel, which provides an artificial electron channel toward the oxic zone (Matturro et al., 2017).

An indirect approach for electron transfer is based on bioelectrochemical (BES) systems, such as microbial fuel cells (MFC), microbial electrolysis cells (MEC) and their sediment variants (sMFC and sMEC) (Pisciotta and Dolceamore, 2016). In MFCs, microbes oxidize the organic pollutants and donate electrons to the anode generating current through an external circuit (Morris et al., 2009). In contrast, MEC requires an input of electrical energy to promote reactions otherwise not taking place. MFC might play a dual role; it can serve as an electron acceptor at the anode and produce energy as electric current. The BES systems usually require exoelectrogens microbes, such as *G. metallireducens* or *Shewanella oneidensis*, capable of extracellular electron transfer (Pisciotta and Dolceamore, 2016). Nevertheless, the anodic microbial communities used to be adapted to the pollutants and environmental conditions, including the externally added surfactants (Li et al., 2018).

Various omics approaches can be used for studying the composition of the microbial communities, metabolic interactions and networks formed during interspecies electron transfer. For instance, a transcriptomic study showed the importance of pili, flagella and a cytochrome type-c protein during DIET in *G. metallireducens/G. sulfurreducens* co-cultures. The authors also observed different gene expression patterns between DIET and hydrogen- coupled MIET (Shrestha et al., 2013). A metatranscriptomic analysis further proved the DIET between *Geobacter* and *Methanothrix* spp (Holmes et al., 2017). A recent review summarizes how DIET can be detected with meta-omics techniques and also implies the importance of combining omics with other methods to evaluate the metabolic network (Van Steendam et al., 2019). With the combination of genomics and metagenomics with substrate turnover measurements, a new model for energy conservation in cable bacteria was proposed (Müller et al., 2020).

## Systems Biology and Metabolic Engineering

Systems biology integrates the multi-omics data to analyze and build models of complex biological systems, such as microbial consortia; thus, their metabolic networks can be examined as a whole. The use of systems biology approaches in bioremediation is indispensable (Dangi et al., 2019; Jaiswal et al., 2019). Nonetheless, considering only the biological aspects of bioremediation is not enough for a successful treatment. Besides modeling cellular and community functions, geochemical and ecological models of the site should be built in an environmental systems biology approach (Hazen, 2019). The knowledge on metabolic networks allows us to fine-tune the processes in the network. Employing metabolic engineering, we can add delete or duplicate genes or entire gene clusters, alter the regulation of pathways or stimulate pathways with precursors, thus tailor a better, more efficient system (Dangi et al., 2019). Metabolic engineering is a relatively young concept; therefore, it is yet rarely applied in anaerobic hydrocarbon biodegradation. In a recent study, a broad-host-range benzoyl-CoA expression cassette was developed for benzoate biodegradation, which might work in the original host or can also be naturally transferred into the indigenous microbes of the contaminated site (Zamarro et al., 2017). Stimulating metabolic pathways is the other aspect of metabolic engineering. The stimulating effect of activated carbon on anaerobic naphthalene biodegradation was recently demonstrated. Besides, the authors hypothesized that activated carbon also stimulates the DIET (Bonaglia et al., 2020). Stimulating DIET by electroconductive materials may be the driving force of anaerobic biodegradation processes (Aulenta et al., 2020).

## Applications and Prospects

Given that hydrocarbons are increasingly spreading into anoxic environments, the necessity of a deeper understanding of the anaerobic degradation pathways is steadily growing.

Investigation of the anaerobic biodegradation of petroleum hydrocarbons has disclosed surprisingly diverse and divergent metabolisms in comparison with aerobic pathways and revealed novel biochemical microbial profiles (Heider et al., 1998). Recent advances widened the scope of anaerobic bioremediation technologies. The exploitation of such degradative capacities may aid in various cases as follows:

(a) Decontamination of anoxic environments, anaerobic treatments of aquifers, oceanic water column, wastewater and sewage sludge. Anaerobic biomineralisation of aliphatic and aromatic compounds is a promising alternative to oxic biodegradation strategies (Holliger and Zehnder, 1996). Because degradation processes under anaerobic conditions are acknowledged to be less efficacious and slower than under aerobic conditions, this is only appropriate at contaminated sites with low air contact or which are barely aerated (Heider et al., 1998). The processes can be accelerated by adding external electron acceptor molecules such as nitrate, sulfate, etc., but these might be quite costly solutions and have severe environmental impacts.

(b) Bioenergy recovery. *G. metallireducens* GS-15 is a toluene-degrading strain under iron-reducing conditions that was proven to be also an “electricigen.” In other words, this strain may be utilized for low-power generation (Lovley, 2006; Foght, 2008). Syntrophic microbial communities can convert contaminants into methane. This process can also be a promising tool in microbial energy recovery, but unfortunately, it is a relatively slow process (Suflita et al., 2004; Gieg et al., 2008; Gray et al., 2010; Xia et al., 2016). Another promising approach to recover energy derived from the oxidation of organic pollutants applies BES-coupled, especially MFC-coupled bioremediation technologies (Morris et al., 2009; Pisciotta and Dolceamore, 2016; Li et al., 2018). MFCs have dual advantages: (i) the anode can serve as an electron acceptor and thus accelerates the bioremediation processes; (ii) it is integrated into an electric circuit to generate electric current. A severe technical limitation of this approach is to construct an electric network covering large contaminated areas.

(c) The remarkable diversity of biological catalysts involved in the anaerobic metabolism of hydrocarbons offers a broader range of applications by overcoming several limiting abiotic and biotic factors. The use of isolated enzymes mitigates microbial competitiveness, limiting nutrients, etc. However, the biocatalyst approach is still costly (low yield of enzyme production), and the enzyme integrity needs to be improved (Setti et al., 1997; Sutherland et al., 2004; Alcalde et al., 2006; Peixoto et al., 2011).

Many new insights are needed to provide improvements to anaerobic biodegradation treatments. Physicochemical characterization of the polluted sites is a critical step for the application of the most efficacious approach. However, characterization of the natural local microflora is at least as necessary. Employing multi-omics techniques, the composition of the microbial community and the metabolic interactions of the community members, as well as other organisms, such as plants, can be discovered. So, cost-effective strategies can be planned for the remediation of hydrocarbon-contaminated sites. The recovery and the characterization of potential hydrocarbon degraders (in pure and mixed cultures) are important to deepen the understanding of their metabolic pathways, for the enhancement of the bioremediation efficiency and identification of interesting functional genes. In many cases, the microbes actively participating in biodegradation can not be isolated and cultured; however, the omics techniques enable us to characterize the metabolic activities of environmental samples which can be further used as inoculum in other remediation projects.

Furthermore, omics techniques allow us to study the biodegradation processes at the system level, metabolic blocks, “metabolic bricks” can be generated and used in metabolic engineering processes for the construction of targeted or multipurpose strains/microbial consortia.

The horizontal gene transfer events taking place in the environment might spread these metabolic bricks to the indigenous microbes and thus increasing the lifetime of a newly introduced metabolic route in the environment.

## Conclusion

The multi-omics era provides a multitude of opportunities for the examination of individual microbes, microbial communities, their metabolism and interactions. Genomics and metagenomics characterize the metabolic potential of the microorganisms; we can identify new microbes in a community and elucidate new pathways and genes which are responsible for hydrocarbon biodegradation. Transcriptomics and metatranscriptomics can further deepen our knowledge by highlighting active pathways in a genome or metagenome under given circumstances. It can even elucidate the role of less abundant members of a microbial community, and it highlights the importance of metabolic networks between community members. Similar results are provided by proteomic and metaproteomic studies, while metabolomics will confirm the chemical reactions occurring during hydrocarbon biodegradation and anaerobic respiration. The omics approaches, alone or in combination, are powerful tools to characterize microbial communities of hydrocarbon-contaminated sites and see the whole network as one big picture. Characterization of the microbiome at polluted sites is a crucial step for the application of the most efficacious remediation approaches. Based on the data provided by omics approaches, cost-effective strategies can be planned for the remediation of hydrocarbon-contaminated sites. The recovery and characterization of potential hydrocarbon-degraders (in pure and mixed cultures) are essential to broaden our understanding of their metabolic pathways, in order to enhance bioremediation efficiency and to identify interesting functional genes. Moreover, in combination with genetic/genomic/metabolic engineering, the stability, the catalytic potential and the enzyme production yield may be increased to promote successful bioremediation strategies. Most of the contaminants are mixtures of various hazardous materials; therefore, complex metabolic activities are required for the complete elimination of these pollutants. The revolution of the new high throughput technologies enables to study the molecular events at the system level and to design synthetic microbiome for targeted challenges, to combine remediation to energy recovery and to redirect the processes from simple elimination of pollutants to production of added-value materials (De Vrieze et al., 2018).

## Author Contributions

KL wrote the dominant part of the draft manuscript including the chapters on omics technologies. ÁEK and ÁS contributed to the section of “Introduction,” NB and GV were involved in the section of “Electron transfer between bacterial cells during hydrocarbon biodegradation.” TK wrote the draft version of the section “Applications and prospects,” AB and KP contributed to the part of “Metabolic aspects of hydrocarbon biodegradation, KP provided input to the sections “Introduction” and “Phytomicroremediation: plant-microbe, microbe-microbe interactions,” GR contributed to the part of “Syntrophy in hydrocarbon-degrading microbial communities,” “Phytomicroremediation: plant-microbe, microbe-microbe interactions and Conclusion.” KL, GR, and KP conceptualized the manuscript. GR and KP provided critical feedback and made the final improvement of the manuscript. All authors contributed to the figures, tables, and literature search and contributed to the article and approved the submitted version.

## Conflict of Interest

TK was employed by the company Enviroinvest Zrt. The remaining authors declare that the research was conducted in the absence of any commercial or financial relationships that could be construed as a potential conflict of interest.
